# Mr. Earl's Invention of an Aneurismal Needle

**Published:** 1811-01

**Authors:** Henry Earle

**Affiliations:** Hanover Square


### To the Editors of the Medical and Physical Journal.

### Mr. Earl's Invention of an Aneurismal Needle.

GENTLEMEN,

IN some Surgical observations which have been lately published by Mr. Ramsden, under the head of "Cases of Aneurism with practical Remarks," will be found a description of two needles; the first is said to be the invention of Mr. J. I. Watt, the second of Mr. Henry Earle. In referring to the plates, the reader will no doubt be much surprised and disappointed to find, nearly the same instrument described as the invention of two different people. It must naturally strike him that the second has been borrowed from the first without any acknowledgment on the part of its pretended author. It is to remove any such impression, which cannot fail of being injurious to my reputation, that I beg the admission of these lines into your widely circulating Journal. The real fact will be found to be, that they were both invented at the same time, and both by me, as I trust I shall prove in a most satisfactory manner.

The circumstances which gave rise to their invention were as follow. A case of axillary aneurism occurred in St. Bartholomew's Hospital, in which Mr. Ramsden took up the subclavian artery just after it emerges from behind the anterior scalenus muscle; the aneurismal tumour was very large, and had elevated the clavicle considerably higher than natural. The artery was found without any difficulty, but the operation was nearly foiled at a point which was little expected. In consequence of the clavicle being pushed up, and the depth of the wound, it was found impossible to pass a ligature round the artery, notwithstanding Mr. Ramsden had prepared himself with a great variety of needles. Though these were different in shape, they were similar in principle, and that unfortunately was imperfect. I cannot forbear here remarking, that it is to be lamented that Surgeons do not pay more attention to this mechanical part of their profession; the fabrication and invention of instruments is left almost entirely to artists, who must be ignorant of the object to be attained; their instruments appear very eligible in theory, but when submitted to the test of experiment they will too often be found insufficient and useless.

For above an hour and half, all attempts to pass a ligature were ineffectual, though needles were several times passed under the artery so as to appear on the opposite side; further they could not pass, it being impossible to depress the handle in order to raise the point. Another fault which these needles had, had, was the length of the eye, whereby the ligature remained on one side of the artery when the point was on the other. At length I handed to Mr. Ramsden part of the stilet of a flexible metallic catheter, which I had flattened for the purpose. This rude instrument, from its great pliability, passed readily under the artery and was drawn up on the opposite side, a ligature was then fastened to it, and the operation happily concluded.

During all this time I was close to the patient, and had an opportunity of observing the cause of embarrassment and the means whereby it might be removed. That night I sketched the instruments which have been described by Mr. R. and the following morning directed them to be made by Mr. M'Lellan, an ingenious instrument maker at St. Bartholomew's. The first that was completed consisted of a flat silver cannula slightly curved at its lower extremity, with two small wings at its upper, at the back there was a groove for the ligature to lie in. In this cannula was fitted a spring about an inch and half longer than it, at the upper extremity of which there was a small handle, at the lower an eye for the ligature. When this instrument is to be used, it must be armed with a ligature which is to be placed in the groove and attached to the wings at the top, the spring being drawn within the cannula. It is then to be passed under the edge of the artery, the ligature loosed and the handle depressed, when the spring, from its elasticity, will rise on the opposite side, carrying with it the ligature, which must be detached and the spring drawn within the cannula, or the needle may be threaded after it has passed under the artery.

This instrument I shewed to Mr. Ramsden, and at his request gave him the preceding account. I also showed it to Mr. Astley Cooper, who received it with the liberality and zeal with which he never fails to embrace any thing that is likely to improve the Profession. On trying this needle on the dead subject I found that the spring in its passage formed too large a circle, whereby the neighbouring nerves were endangered. I recurred, therefore, to my original plan, and ordered a needle of soft metal about an inch and half long to be made to fit the cannula, into the upper part of which was fitted a flat steel spring of the same length as the cannula, having a handle at the top, which when depressed would push out the needle with its ligature. In short, I ordered the precise needle which has been described as Mr. Watt's contrivance. This instrument I showed to Mr. Ramsden and several other gentlemen, and it was publicly sold at M'Lellan's as my invention. In the mean time Mr. Watt invented a needle which was made at the same shop, the object of which was the same as mine, though the means were materially terially different. This he afterwards abandoned, for reasons best known to himself, and he did me the honour of adopting mine. The only alteration he made was substituting a steel needle for the flexible one, and not continuing the slit up the whole length of the cannula. I need no other proof of the truth of this statement than the joint testimony of the instrument maker and his man. The reason of my intruding myself in this manner is to clear my character from the stigma which might be cast on it of being an unmeaning copyist. If my name had not been mentioned at all, Mr. Watt should have been most welcome to what little credit may attach to the inventor of such an instrument; but as it has appeared before the public, I certainly wish that things should be represented in their true light. I am also desirous of making this statement to avoid the imputation of having borrowed from the French, without making due acknowledgment. I feel myself placed in a particularly unpleasant situation, for at the very time that I am attempting to prove that the instrument has been taken from me, I am aware, that I may be accused of having copied from Desault. Since I invented this, to me, unfortunate needle, I have read a description of one nearly similar in Desault's works edited by Bichat. I have stated the circumstances which gave rise to *my* instrument; which was the genuine offspring of necessity, fostered by a little turn for mecanics which I have always endeavoured to use for the advantage of my Profession. Similiar causes might have produced the one described by Bichat. But with Mr. Watt and myself circumstances are widely different. He daily saw and examined the instrument which I had invented, whilst he was superintending the making of his own. It cannot, I think, for a minute, be supposed that I should attempt so gross an imposition, as to endeavour to pass for my own, an instrument which I knew to be the invention of another; particularly, when I should be liable to be detected by every person who perused the works of that excellent practitioner Desault. As I have now discovered a prior author, I shall cede all claim to originality, and deem myself fortunate in introducing into this country an instrument which is likely to be of practical utility. I cannot conclude without thanking Mr. Ramsden for the very unmerited encomium he has been pleased to pass on me, and stating that I am perfectly convinced that had he been aware of the facts which I have mentioned, he would have rendered all this egotism unnecessary. To avoid any further altercation or paper war on a subject which can only be interesting to individuals, I can produce the evidence of Mr. M'Lellan to the veracity of the above account.

*Hanover Square, December* 10, 1810. HENRY EARLE.

